# Model Selection in a Composite Likelihood Framework Based on Density Power Divergence

**DOI:** 10.3390/e22030270

**Published:** 2020-02-27

**Authors:** Elena Castilla, Nirian Martín, Leandro Pardo, Konstantinos Zografos

**Affiliations:** 1 Interdisciplinary Mathematics Institute and Department of Statistics and O.R. I, Complutense University of Madrid, 28040 Madrid, Spain; lpardo@mat.ucm.es; 2 Interdisciplinary Mathematics Institute and Department of Financial and Actuarial Economics & Statistics, Complutense University of Madrid, 28003 Madrid, Spain; nirian@estad.ucm.es; 3 Department of Mathematics, University of Ioannina, 45110 Ioannina, Greece; kzograf@uoi.gr

**Keywords:** composite likelihood, composite minimum density power divergence estimators, model selection

## Abstract

This paper presents a model selection criterion in a composite likelihood framework based on density power divergence measures and in the composite minimum density power divergence estimators, which depends on an tuning parameter α. After introducing such a criterion, some asymptotic properties are established. We present a simulation study and two numerical examples in order to point out the robustness properties of the introduced model selection criterion.

## 1. Introduction

Composite likelihood inference is an important approach to deal with those real situations of large data sets or very complex models, in which classical likelihood methods are computationally difficult, or even, not possible to manage. Composite likelihood methods have been successfully used in many applications concerning, for example, genetics ([1]), generalized linear mixed models ([2]), spatial statistics ([3,4,5]), frailty models ([6]), multivariate survival analysis ([7,8]), etc.

Let us introduce the problem, adopting here the notation by [9]. Let $\left\{f\left(\cdot ;\theta \right),\theta \in \Theta \subseteq R^{p},p\geq 1\right\}$ be a parametric identifiable family of distributions for an observation $y={\left(y_{1},\ldots ,y_{m}\right)}^{T}$, a realization of a random *m*-vector Y. In this setting, the composite likelihood function based on *K* different marginal or conditional distributions has the form

$$\mathrm{CL}\left(\theta ,y\right)=\prod_{k=1}^{K}{\left(f_{A_{k}}\left(y_{j},j\in A_{k};\theta \right)\right)}^{w_{k}}$$

and the corresponding composite log-density

(1) $$\log\mathrm{CL}\left(\theta ,y\right)=\sum_{k=1}^{K}w_{k}\ell_{A_{k}}\left(\theta ,y\right),$$

with $\ell_{A_{k}}\left(\theta ,y\right)=\log f_{A_{k}}\left(y_{j},j\in A_{k};\theta \right)$, where ${\left\{A_{k}\right\}}_{k=1}^{K}$ is a family of sets of indices associated either with marginal or conditional distributions involving some $y_{j}$, j∈{1,…,m} and $w_{k}$, k=1,…,K are non-negative and known weights. If the weights are all equal, then they can be ignored. In this case, all the statistical procedures give equivalent results. The composite maximum likelihood estimator (CMLE), ${\hat{\theta }}_{c}$, is obtained by maximizing, in respect to θ∈Θ, the expression (1).

The CMLE is consistent and asymptotically normal and, based on it, we can establish hypothesis testing procedures in a similar way to the classical likelihood ratio test, Wald test or Rao’s score test. A development of the asymptotic theory of the CMLE including its application to obtain the composite ratio statistics, Wald-type tests and Rao score tests in the context of composite likelihood can be seen in [10]. However, in [11,12,13] is shown that the CMLE and the derived testing procedures present an important lack of robustness. In this sense, [11,12,13] derived some new distance-based estimators and tests with good robustness behaviour without an important loss of efficiency. In this paper, we are going to consider the composite minimum density power divergence estimator (CMDPDE), introduced in [12], in order to present a model selection criterion in a composite likelihood framework.

Model selection criteria, for summarizing data evidence in favor of a model, is a very well studied subject in statistical literature, overall in the context of full likelihood. The construction of such criteria requires a measure of similarity between two models, which are typically described in terms of their distributions. This can be achieved if an unbiased estimator of the expected overall discrepancy is found, which measures the statistical distance between the true, but unknown model, and the entertained model. Therefore, the model with the smallest value of the criterion is the most preferable model. The use of divergence measures, in particular Kullback–Leibler divergence ([14]), to measure this discrepancy, is the main idea of some of the most known criteria: Akaike Information Criterion (AIC, [15,16]), the criterion proposed by Takeuchi (TIC, [17]) and other modifications of AIC [18]. DIC criterion, based on the density power divergence (DPD), was presented in [19] and, recently, [20] presented a local BHHJ power divergence information criterion following [21]. In the context of the composite likelihood there are some criteria based on Kullback–Leibler divergence, see for instance [22,23,24] and references therein. To the best of our knowledge only Kullback–Leibler divergence was used to develop model selection criteria in a composite likelihood framework. To fill this gap, our interest is now focused on DPD.

In this paper, we present a new information criterion for model selection in the framework of composite likelihood based on DPD measure. This divergence measure, introduced and studied in the case of complete likelihood by [25], has been considered previously in [12,13] in the context of composite likelihood. In these papers, a new estimator, the CMDPDE, was introduced and its robustness in relation to the CMLE as well as the robustness of some families of test statistics were studied, but the problem of model selection was not considered. This problem is considered in this paper. The criterion introduced in this paper will be called composite likelihood DIC criterion (CLDIC). The motivation of considering a criterion based on DPD instead of Kullback–Leibler divergence is due to the robustness of the procedures based on DPD in statistical inference, not only in the context of full likelihood [25,26], but also in the context of composite likelihood [12,13]. In Section 2, the CMDPDE is presented and some properties of this estimator are discussed. The new model selection criterion, CLDIC, based on CMDPDE is introduced in Section 3 and some of its asymptotic properties are studied. A simulation study is carried out in Section 4 and some numerical examples are presented in Section 5. Finally, some concluding remarks are presented in Section 6.

## 2. Composite Minimum Density Power Divergence Estimator

Given two probability density functions *g* and *f*, associated with two *m*-dimensional random variables respectively, the DPD ([25]) measures a statistical distance between *g* and *f* by

(2) $$\begin{array}{c} d_{\alpha }\left(g,f\right)=\int_{R^{m}}\left\{f{\left(y\right)}^{1+\alpha }-\left(1+\frac{1}{\alpha }\right)f{\left(y\right)}^{\alpha }g\left(y\right)+\frac{1}{\alpha }\;g{\left(y\right)}^{1+\alpha }\right\}dy, \end{array}$$

for α>0, while for α=0 it is defined by

$$d_{0}\left(g,f\right)=\lim_{\alpha \to 0^{+}}d_{\alpha }\left(g,f\right)=d_{KL}\left(g,f\right),$$

where $d_{KL}\left(g,f\right)$ is the Kullback–Leibler divergence (see, for example, [26]). For α=1, the expression (2) leads to the $L_{2}$ distance $L_{2}\left(g,f\right)=\int_{R^{m}}{\left(f(y)-g(y)\right)}^{2}dy.$ It is also interesting to note that (2) is a special case of the so-called Bregman divergence

(3) $$\int_{R^{m}}\left[T\left(g\left(y\right)\right)-T\left(f\left(y\right)\right)-\{g\left(y\right)-f\left(y\right\}T^{\prime }\left(f\left(y\right)\right)\right]dy.$$

If we consider $T\left(l\right)=\frac{1}{\alpha }l^{1+\alpha }$ in (3), we get $d_{\alpha }\left(g,f\right)$. The parameter α controls the trade-off between robustness and asymptotic efficiency of the parameter estimates which are the minimizers of this family of divergences. For more details about this family of divergence measures we refer to [27].

Let now $Y_{1},\ldots ,Y_{n}$ be independent and identically distributed replications of Y which are characterized by the true but unknown distribution *g*. Taking into account that the true model *g* is unknown, suppose that $\Xi =\{f\left(\cdot ;\theta \right),\theta \in \Theta \subseteq R^{p},p\geq 1\}$ is a parametric identifiable family of candidate distributions to describe the observations $y_{1},\ldots ,y_{n}$. Then, the DPD between the true model *g* and the composite likelihood function, CL(θ,·), associated to the parametric model f(·;θ) is defined as

(4) $$\begin{array}{cc} d_{\alpha }\left(g\left(\cdot \right),\mathrm{CL}\left(\theta ,\cdot \right)\right)= & \int_{R^{m}}\left\{\mathrm{CL}{\left(\theta ,y\right)}^{1+\alpha }-\left(1+\frac{1}{\alpha }\right)\mathrm{CL}{\left(\theta ,y\right)}^{\alpha }g\left(y\right)+\frac{1}{\alpha }g{\left(y\right)}^{1+\alpha }\right\}dy, \end{array}$$

for α>0, while for α=0 we have $d_{KL}\left(g\left(\cdot \right),\mathrm{CL}\left(\theta ,\cdot \right)\right)$, which is defined by

(5) $$d_{KL}\left(g\left(\cdot \right),\mathrm{CL}\left(\theta ,\cdot \right)\right)=\int_{R^{m}}g\left(y\right)\log\frac{g(y)}{\mathrm{CL}(\theta ,y)}dy.$$

In Section 3, we are going to introduce and study the CLDIC criterion based on (4).

Let

(6) $${\left\{M_{k}\right\}}_{k\in \left\{1,\ldots ,\ell \right\}}$$

be a family of candidate models to govern the observations $Y_{1},\ldots ,Y_{n}$. We shall assume that the true model is included in ${\left\{M_{k}\right\}}_{k\in \left\{1,\ldots ,\ell \right\}}.$ For a specific k=1,…,ℓ, the parametric model $M_{k}$ is described by the composite likelihood function

$$\mathrm{CL}\left(\theta ,\cdot \right),\;\;\theta \in \Theta_{k}\subset R^{k}.$$

In this setting, it is quite clear that the most suitable candidate model to describe the observations is the model that minimizes the DPD in (4). However, the unknown parameter θ is included in it, so it is not possible to use directly this measure for the choice of the most suitable model. A way to overcome this problem is to plug-in, in (4), the unknown parameter θ by an estimator which is desirable to obey some nice properties, like consistency and asymptotic normality. Based on this point, the CMDPDE, introduced in [12], can be used. This estimator is described in the sequel for the sake of completeness.

If we denote the kernel of (4) as

(7) $$W_{\alpha }\left(\theta \right)=\int_{R^{m}}\mathrm{CL}{\left(\theta ,y\right)}^{1+\alpha }dy-\left(1+\frac{1}{\alpha }\right)\int_{R^{m}}\mathrm{CL}{\left(\theta ,y\right)}^{\alpha }g\left(y\right)dy,$$

we can write

$$d_{\alpha }\left(g\left(\cdot \right),\mathrm{CL}\left(\theta ,\cdot \right)\right)=W_{\alpha }\left(\theta \right)+\frac{1}{\alpha }\int_{R^{m}}g{\left(y\right)}^{1+\alpha }dy$$

and the term $\frac{1}{\alpha }\int_{R^{m}}g{\left(y\right)}^{1+\alpha }dy$ does not depend on θ and could be ignored in (9). A natural estimator of $W_{\alpha }\left(\theta \right)$, given in (7), can be obtained by observing that the last integral in (7), can be expressed in the form $\int_{R^{m}}\mathrm{CL}{\left(\theta ,y\right)}^{\alpha }dG\left(y\right)$, for *G* the distribution function corresponding to *g*. Hence, if the empirical distribution function of $Y_{1},\ldots ,Y_{n}$ will be exploited, this last integral is approximated by $\frac{1}{n}\sum_{i=1}^{n}\mathrm{CL}{\left(\theta ,Y_{i}\right)}^{\alpha }$, i.e.,

(8) $$W_{n,\alpha }\left(\theta \right)=\int_{R^{m}}\mathrm{CL}{\left(\theta ,y\right)}^{\alpha +1}dy-\left(1+\frac{1}{\alpha }\right)\frac{1}{n}\sum_{i=1}^{n}\mathrm{CL}{\left(\theta ,Y_{i}\right)}^{\alpha }.$$

**Definition** **1.** *The CMDPDE of **θ**, ${\hat{\theta }}_{c}^{\alpha },$ is defined, for α>0, by*

(9) $${\hat{\theta }}_{c}^{\alpha }=\arg\min_{\theta \in \Theta }W_{n,\alpha }\left(\theta \right).$$

We shall denote the score of the composite likelihood by

(10) $$u\left(\theta ,y\right)=\frac{\partial log\mathrm{CL}(\theta ,y)}{\partial \theta }.$$

Let $\theta_{0}$ be the true value of the parameter θ. In [12], it was shown that the asymptotic distribution of ${\hat{\theta }}_{c}^{\alpha }$ is given by

$$\sqrt{n}\left({\hat{\theta }}_{c}^{\alpha }-\theta_{0}\right)\underset{n\to \infty }{\overset{L}{⟶}}N\left(0_{p},H_{\alpha }{\left(\theta_{0}\right)}^{-1}J_{\alpha }\left(\theta_{0}\right)H_{\alpha }{\left(\theta_{0}\right)}^{-1}\right),$$

being

(11) $$H_{\alpha }\left(\theta \right)=\int_{R^{m}}\mathrm{CL}{\left(\theta ,y\right)}^{\alpha +1}u\left(\theta ,y\right)u{\left(\theta ,y\right)}^{T}dy$$

and

(12) $$\begin{array}{cc} J_{\alpha }\left(\theta \right) & =\int_{R^{m}}\mathrm{CL}{\left(\theta ,y\right)}^{2\alpha +1}u\left(\theta ,y\right)u{\left(\theta ,y\right)}^{T}dy\\ \; & \;-\int_{R^{m}}\mathrm{CL}{\left(\theta ,y\right)}^{\alpha +1}u\left(\theta ,y\right)dy\int_{R^{m}}u{\left(\theta ,y\right)}^{T}\mathrm{CL}{\left(\theta ,y\right)}^{1+\alpha }dy. \end{array}$$

**Remark** **1.** *For α=0 we get the CMLE of **θ***

(13) $${\hat{\theta }}_{c}=\arg\min_{\theta \;\in \Theta }\left(-\frac{1}{n}\sum_{i=1}^{n}\log\mathrm{CL}\left(\theta ,y_{i}\right)\right).$$

*At the same time it is well-known that*

$$\sqrt{n}\left({\hat{\theta }}_{c}-\theta \right)\underset{n\to \infty }{\overset{L}{⟶}}N\left(0_{p},G_{*}{\left(\theta \right)}^{-1}\right),$$

*where $G_{*}\left(\theta \right)$ denotes the Godambe information matrix defined by $G_{*}\left(\theta \right)=H\left(\theta \right)J{\left(\theta \right)}^{-1}H\left(\theta \right)$, with H(θ) being the sensitivity or Hessian matrix and J(θ) being the variability matrix, defined, respectively, by*

$$\begin{array}{c} H\left(\theta \right)=E_{\theta }\left[-\frac{\partial }{\partial \theta }u{\left(\theta ,y\right)}^{T}\right],\;J\left(\theta \right)=E_{\theta }\left[u\left(\theta ,y\right)u{\left(\theta ,y\right)}^{T}\right]. \end{array}$$

## 3. A New Model Selection Criterion

In order to describe the CLDIC criterion we consider the model $M_{k}$ given in (6). Following standard methodology (cf. [28], pp. 240), the most suitable candidate model to describe the data $Y_{1},\ldots ,Y_{n}$ is the model that minimizes the expected estimated DPD

(14) $$E_{Y_{1},\ldots ,Y_{n}}\left[d_{\alpha }\left(g\left(\cdot \right),\mathrm{CL}\left({\hat{\theta }}_{c}^{\alpha },\cdot \right)\right)\right],$$

subject to the assumption that the unknown model *g* is belonging to Ξ, i.e., the true model is included in ${\left\{M_{s}\right\}}_{s\in \left\{1,\ldots ,\ell \right\}}$ and taking into account that ${\hat{\theta }}_{c}^{\alpha }$, defined in (9), is a consistent and asymptotic normally distributed estimator of θ. However, this expected value is still depending on the unknown parameter θ. So, as a criterion, it should be used an asymptotically unbiased estimator of (14), for g∈Ξ.

The most appropriate model to select is the model which minimizes the expected value

$$E_{Y_{1},\ldots ,Y_{n}}\left[W_{\alpha }\left({\hat{\theta }}_{c}^{\alpha }\right)\right].$$

This expected value is still depending on the unknown parameter θ. So, an asymptotically unbiased estimator of the above expected value could be the basis of a selection criterion, for g∈Ξ. In order to proceed with the derivation of such an asymptotically unbiased estimator of $E_{Y_{1},\ldots ,Y_{n}}\left[W_{\alpha }\left({\hat{\theta }}_{c}^{\alpha }\right)\right]$. The empirical version of $W_{\alpha }\left(\theta \right)$, in (7), is $W_{n,\alpha }\left(\theta \right)$, given in (8), and plays a central role in the development of the model selection criterion on the basis of the next theorem which expresses the expected value $E_{Y_{1},\ldots ,Y_{n}}\left[W_{\alpha }\left({\hat{\theta }}_{c}^{\alpha }\right)\right]$ by means of the respective expected value of $W_{n,\alpha }\left({\hat{\theta }}_{c}^{\alpha }\right)$, in an asymptotically equivalent way.

**Theorem** **1.** *If the true distribution g belongs to the parametric family Ξ and $\theta_{0}$ denotes the true value of the parameter **θ**, then we have*

$$E_{Y_{1},\ldots ,Y_{n}}\left[W_{\alpha }\left({\hat{\theta }}_{c}^{\alpha }\right)\right]=E_{Y_{1},\ldots ,Y_{n}}\left[W_{n,\alpha }\left({\hat{\theta }}_{\alpha }\right)+\frac{\alpha +1}{n}trace\left(J_{\alpha }\left(\theta_{0}\right)H_{\alpha }{\left(\theta_{0}\right)}^{-1}\right)\right]+o_{p}\left(1\right)$$

*with $H_{\alpha }\left(\theta \right)$ and $J_{\alpha }\left(\theta \right)$ given in (11) and (12), respectively.*

Based on the above theorem, the proof of which is presented in a full detail in the Appendix A, an asymptotic unbiased estimator of $E_{Y_{1},\ldots .,Y_{n}}\left[W_{\alpha }\left({\hat{\theta }}_{c}^{\alpha }\right)\right]$ is given by

$$W_{n,\alpha }\left({\hat{\theta }}_{c}^{\alpha }\right)+\frac{\alpha +1}{n}trace\left(J_{\alpha }\left({\hat{\theta }}_{c}^{\alpha }\right)H_{\alpha }{\left({\hat{\theta }}_{c}^{\alpha }\right)}^{-1}\right).$$

This ascertainment is the basis and a strong motivation for the next definition which introduces the model selection criterion.

**Definition** **2.** *Let ${\left\{M_{k}\right\}}_{k\in \left\{1,\ldots ,\ell \right\}}$ be candidate models for the observations $Y_{1},\ldots ,Y_{n}$. The selected model $M^{*}$ verifies*

$$M^{*}=\min_{k\in \{1,\ldots ,,\ell \}}CLDIC_{\alpha }\left(M_{k}\right),$$

*where*

$$CLDIC_{\alpha }\left(M_{k}\right)=W_{n,\alpha }\left({\hat{\theta }}_{c}^{\alpha }\right)+\frac{\alpha +1}{n}trace\left(J_{\alpha }\left({\hat{\theta }}_{c}^{\alpha }\right)H_{\alpha }{\left({\hat{\theta }}_{c}^{\alpha }\right)}^{-1}\right),$$

*$W_{n,\alpha }\left(\theta \right)$ was given in (8) and $J_{\alpha }\left(\theta \right)$ and $H_{\alpha }\left(\theta \right)$ were defined in (11) and (12), respectively.*

The next remark summarizes the model selection criterion in the case α=0 and it therefore extends, in a sense, the pioneer and classic AIC.

**Remark** **2.** *For α=0 we have,*

$$d_{KL}\left(g\left(\cdot \right),\mathrm{CL}\left(\theta ,\cdot \right)\right)=W_{0}\left(\theta \right)+\int_{R^{n}}g\left(y\right)\log g\left(y\right)dy$$

*with $W_{0}\left(\theta \right)=-\int_{R^{n}}\log\mathrm{CL}\left(\theta ,y\right)g\left(y\right)dy$. Therefore, the most appropriate model which should be selected, is the model which minimizes the expected value*

(15) $$E_{Y_{1},\ldots ,Y_{n}}\left[W_{0}\left({\hat{\theta }}_{c}\right)\right],$$

*where ${\hat{\theta }}_{c}$ is the CMLE of $\theta_{0}$ defined in (9).*

*The expected value (15) is still depending on the unknown parameter **θ**. A natural estimator of $W_{0}\left({\hat{\theta }}_{c}\right)$ can be obtained by replacing the distribution function G, of g, by the empirical distribution function based on $Y_{1},\ldots ,Y_{n}$,*

$$W_{n,0}\left(\theta \right)=-\frac{1}{n}\sum_{i=1}^{n}\log\mathrm{CL}\left(\theta ,y_{i}\right).$$

*Based on it, we select the model $M^{*}$ that verifies*

$$M^{*}=\min_{k\in \{1,\ldots ,\ell \}}CLDIC_{0}\left(M_{k}\right),$$

*with*

$$CLDIC_{0}\left(M_{k}\right)=H_{n,0}\left({\hat{\theta }}_{c}\right)+\frac{1}{n}trace\left(J\left({\hat{\theta }}_{c}\right)H{\left({\hat{\theta }}_{c}\right)}^{-1}\right),$$

*where $J({\hat{\theta }}_{c})$ and $H({\hat{\theta }}_{c})$ are defined in Remark 1. In a manner, quite similar to that of the previous theorem, it can be established that $CLDIC_{0}\left(M_{k}\right)$ is an asymptotic unbiased estimator of $E_{Y_{1},\ldots ,Y_{n}}\left[W_{0}\left({\hat{\theta }}_{c}\right)\right].$*

*This would be the model selection criterion in a composite likelihood framework based on Kullback–Leibler divergence. We can observe that this criterion coincides with the criterion given in [22] as a generalization of the classical criterion of Akaike, which will be referred from now as Composite Akaike Information Criterion (CAIC).*

## 4. Numerical Simulations

### 4.1. Scenario 1: Two-Component Mixed Model

We are starting with a simulation example, which is motivated and follows ideas from the paper [29] and the Example 4.1 in [20] which will compare the behaviour of the proposed criteria with the CAIC criterion, for α=0 (see Remark 2).

Consider the random vector $Y={\left(Y_{1},Y_{2},Y_{3},Y_{4}\right)}^{T}$ from an unknown density *g* and let now $Y_{1},\ldots ,Y_{n}$ be independent and identically distributed replications of Y which are described by the true but unknown distribution *g*. Taking into account that the true model *g* is unknown, suppose that $\left\{f\left(\cdot ;\theta \right),\theta \in \Theta \subseteq R^{p},p\geq 1\right\}$ is a parametric identifiable family of candidate distributions to describe the observations $y_{1},\ldots ,y_{n}$. Let also CL(θ,y) denotes the composite likelihood function associated to the parametric model f(·;θ).

We consider the problem of choosing (on the basis of *n* independent and identically distributed replications $y_{1},\ldots ,y_{n}$ of $Y={\left(Y_{1},Y_{2},Y_{3},Y_{4}\right)}^{T}$) between a 4-variate normal distribution, $N\left(\mu^{N},\Sigma \right)$, with $\mu^{N}={\left(\mu_{1}^{N},\mu_{2}^{N},\mu_{3}^{N},\mu_{4}^{N}\right)}^{T}$ and

$$\Sigma =\left(\begin{array}{cccc} 1 & \rho & 2\rho & 2\rho \\ \rho & 1 & 2\rho & 2\rho \\ 2\rho & 2\rho & 1 & \rho \\ 2\rho & 2\rho & \rho & 1 \end{array}\right),$$

and a 4-variate *t*-distribution with ν degrees of freedom, $t_{\nu }\left(\mu^{t_{\nu }},\Sigma^{*}\right)$, with different location parameters $\mu^{t_{\nu }}={\left(\mu_{1}^{t_{\nu }},\mu_{2}^{t_{\nu }},\mu_{3}^{t_{\nu }},\mu_{4}^{t_{\nu }}\right)}^{T}$ and same variance-covariance matrix Σ, and density,

$$C_{m}{\left|\Sigma^{*}\right|}^{-1/2}{\left[1+\frac{1}{\nu }{\left(y-\mu^{t_{\nu }}\right)}^{T}{\left(\Sigma^{*}\right)}^{-1}\left(y-\mu^{t_{\nu }}\right)\right]}^{-(\nu +m)/2},$$

with $\Sigma^{*}=\frac{\nu -2}{\nu }\Sigma$, $C_{m}={\left(\pi \nu \right)}^{-m/2}\frac{\Gamma [(\nu +m)/2]}{\Gamma (\nu /2)}$ and m=4.

Consider the composite likelihood function,

$$\mathrm{CL}N\left(\rho ,y\right)=f_{A_{1}}^{N}\left(y;\rho \right)f_{A_{2}}^{N}\left(y;\rho \right),$$

with $f_{A_{1}}^{N}\left(y;\rho \right)=f_{12}^{N}\left(y_{1},y_{2};\mu_{1}^{N},\mu_{2}^{N};\rho \right)$ and $f_{A_{2}}^{N}\left(y;\rho \right)=f_{34}^{N}\left(y_{3},y_{4};\mu_{3}^{N},\mu_{4}^{N};\rho \right)$, where $f_{12}^{N}$ and $f_{34}^{N}$ are the densities of the marginals of Y, i.e., bivariate normal distributions with mean vectors ${\left(\mu_{1}^{N},\mu_{2}^{N}\right)}^{T}$ and ${\left(\mu_{3}^{N},\mu_{4}^{N}\right)}^{T}$, respectively, and common variance-covariance matrix

$$\Sigma_{0}=\left(\begin{array}{cc} 1 & \rho \\ \rho & 1 \end{array}\right).$$

In a similar manner consider the composite likelihood

$$\mathrm{CL}t_{\nu }\left(\rho ,y\right)=f_{A_{1}}^{t_{\nu }}\left(y;\rho \right)f_{A_{2}}^{t_{\nu }}\left(y;\rho \right),$$

with $f_{A_{1}}^{t_{\nu }}\left(y;\rho \right)=f_{12}^{t_{\nu }}\left(y_{1},y_{2};\mu_{1}^{t_{\nu }},\mu_{2}^{t_{\nu }};\rho \right)$ and $f_{A_{2}}^{t_{\nu }}\left(y;\rho \right)=f_{34}^{t_{\nu }}\left(y_{3},y_{4};\mu_{3}^{t_{\nu }},\mu_{4}^{t_{\nu }};\rho \right)$, where $f_{12}^{t_{\nu }}$ and $f_{34}^{t_{\nu }}$ are the densities of the marginals of Y, i.e., bivariate *t*-distributions with mean vectors ${\left(\mu_{1}^{t_{\nu }},\mu_{2}^{t_{\nu }}\right)}^{T}$ and ${\left(\mu_{3}^{t_{\nu }},\mu_{4}^{t_{\nu }}\right)}^{T}$, respectively, and common variance-covariance matrix

$$\Sigma_{0}=\left(\begin{array}{cc} 1 & \rho \\ \rho & 1 \end{array}\right).$$

Under this formulation, the simulation study follows in the next two scenarios.

#### 4.1.1. Scenario 1a

Following Example 4.1 in [20], the steps of the simulation study are the following:

- Generate 1000 samples of size n=5,7,10,20,40,50,70,100 from a two component mixture of two 4-variate distributions, namely, a 4-variate normal and a 4-variate *t*-distribution,
  $$h_{\omega }\left(y\right)=\omega N\left(\mu^{N},\Sigma \right)+\left(1-\omega \right)t_{\nu }\left(\mu^{t_{\nu }},\Sigma^{*}\right),\;0\leq \omega \leq 1,$$
  with $\mu^{N}=\left(0,0,0.5,0\right)$ and $\mu^{t_{\nu }}=\left(3.2,1.5,0.5,2\right)$, for ω=0,0.25,0.45,0.5,0.55,0.75,1, ν=5,10,30 degrees of freedom and with specific values of ρ=−0.15,−0.10,0.10. As pointed out in [29], taking into account that Σ should be semi-positive definite, the following condition is imposed: $-\frac{1}{5}\leq \rho \leq \frac{1}{3}$.
- Estimate the common parameter ρ, separately in each model, by using the CMDPDE estimator for different values of the tuning parameter α=0,0.3. The composite density which corresponds to the mixture $h_{\omega }\left(y\right)$ is defined by
  $$\mathrm{CL}\left(\rho ,y\right)=\omega \mathrm{CL}N\left(\rho ,y\right)+\left(1-\omega \right)\mathrm{CL}t_{\nu }\left(\rho ,y\right),\;0\leq \omega \leq 1,$$
  and it is used to obtain the CMDPDE estimator, $\hat{\rho }$, of ρ.
- Define the mixture composite likelihood function
  $$\mathrm{CL}\left(\hat{\rho },y\right)=\omega \mathrm{CL}N\left(\hat{\rho },y\right)+\left(1-\omega \right)\mathrm{CL}t_{\nu }\left(\hat{\rho },y\right),\;0\leq \omega \leq 1.$$
- Calculate $CLDIC_{\alpha }\left(M_{k}\right)$, the value of the model selection criterion considered in this paper, for the two candidate models, with
  $$CLDIC_{\alpha }\left(M_{k}\right)=W_{n,\alpha }\left(\hat{\rho }\right)+\frac{\alpha +1}{n}trace\left(J_{\alpha }\left(\hat{\rho }\right)H_{\alpha }{\left(\hat{\rho }\right)}^{-1}\right).$$
  An explanation of how to obtain this value for the both candidate models is given in Appendix B.
- Compute the times that the 4-variate normal model was selected.

Results are summarized in Table 1. Extreme values of ω=0,1 represent the times that the 4-variate normal model was selected under the 4-variate *t*-distribution and 4-variate normal distribution, respectively. This means that, for ω=1, the perfect discrimination will be achieved when 1000 of the 1000 simulated samples are correctly assigned, while for ω=0, the more near to 0, the better discrimination of the criterion. ω=0.5 means that each sample was generated both from the normal and *t*-distribution in the same proportion.

#### 4.1.2. Scenario 1b

Same Scenario is evaluated under the more-closed means $\mu^{N}=\left(0,1.5,0.5,-0.75\right)$ and $\mu^{t_{\nu }}=\left(0,1.5,0.5,2\right)$ for moderate-large sample sizes and α∈{0,0.2,0.4}. Here ν=5 and ρ=−0.15. Results are shown in Table 2. In this case, the models under consideration are more similar, so it would be understandable that the CLDIC criterion did not discriminate in such as good way.

### 4.2. Scenario 2: Three-Component Mixed Model

Now, we consider a mixed model composed on two 4-variate normal distributions and a 4-variate *t*-distribution with ν=10 degrees of freedom. The three distributions have common variance-covariance matrix, as in the previous scenario, with unknown ρ=−0.15 and different but known means $\mu_{1}^{N}=\left(0,0,0.5,0\right)$, $\mu_{2}^{N}=\left(0,1.5,0.5,0\right)$ and $\mu^{t}=\left(0,1.5,0.5,2\right)$. The model is defined by

$$\omega N\left(\mu_{1}^{N},\Sigma \right)+\lambda N\left(\mu_{2}^{N},\Sigma \right)+\left(1-\omega -\lambda \right)t_{\nu =10}\left(\mu^{t},\Sigma^{*}\right),\;0\leq \omega ,\lambda ,\omega +\lambda \leq 1,$$

with Σ being again a common variance-covariance matrix with unknown parameter ρ of the form

$$\Sigma =\left(\begin{array}{cccc} 1 & \rho & 2\rho & 2\rho \\ \rho & 1 & 2\rho & 2\rho \\ 2\rho & 2\rho & 1 & \rho \\ 2\rho & 2\rho & \rho & 1 \end{array}\right).$$

Following the same steps that in the first scenario, we generate 1000 samples of the three-component mixture for different sample sizes n=5,7,10,20,40,50,70,100 and different values of ω and λ. Then, we consider the problem of choosing among one of the two 4-variate normal distributions and the 4-variate *t*-distribution through the CLDIC criterion, for different values of the tuning parameter α=0,0.3,0.5,0.7. See Table 3 for results. Here, the normal models are denoted by N1 and N2, respectively, while the 4-variate *t*-distribution is denoted by MT. The first three cases evaluate the selected model under these multivariate distributions. In the last two scenarios, a mixed model is considered as the true distribution.

### 4.3. Discussion of Results

In Scenario 1a, two well-differentiated multivariate models are considered. In this case CLDIC criterion works in a very efficient way, with an almost-perfect discrimination for extreme values of ω. The good behaviour is also observed for not so extreme values of ω, such as ω=0.55 or 0.45. We can not observe a significant difference in the choice of α.

In Scenario 1b we consider closer models, which affect the discrimination power of the CLDIC. However, in this case, we do observe great differences when considering different α. While the discrimination power of CLDIC for α=0 (CAIC) and ω=1 is around 75%, for α=0.2 or α=0.4 the behaviour is excellent. This happens also for large but not extreme values of ω, such as ω=0.75. However, a medium value of α turns into a worse discrimination for low values of ω.

Scenario 2 deals with three different models, two multivariate normal and one multivariate *t* (N1, N2 and MT, respectively). The second normal distribution is closer to MT in terms of means. While CLDIC criterion discriminate well between N1 and N2 and between N1 and MT, it has difficulties in distinguishing N2 an MT distributions, overall for small samples sizes and α=0.

It seems, therefore, that when we have well-discriminated models, CLDIC criterion works very well, independently of the sample size and the tuning parameter α considered. Dealing with closer models leads, as expected, to worst results, overall for α=0 (CAIC).

Note that the behaviour of Wald-type and Rao tests based on CMDPDEs was studied in [12,13] through extensive simulation studies.

## 5. Numerical Examples

### 5.1. Choice of the Tuning Parameter

In the previous sections, we have seen that CLDIC criterion works generally very well, independently of α, but that some values present a better behaviour, overall when distinguishing similar models. In these situations, it appears that values close to 0.2 or 0.3 work well, while CAIC criterion presents a worse behaviour. A data-driven approach for the choice of the tuning parameter which would be helpful in practice. The approach of [30] was adapted In [13], for the choice of the optimum α in CMDPDEs. This approach consisted on minimizing the estimated mean squared error by means of a pilot estimator, $\theta^{P}$. This approximation is given by

(16) $${\hat{MSE}}_{\alpha }={\left({\hat{\theta }}_{c}^{\alpha }-\theta^{P}\right)}^{T}\left({\hat{\theta }}_{c}^{\alpha }-\theta^{P}\right)+\frac{1}{n}\mathrm{Trace}\left(H_{\alpha }^{-1}\left({\hat{\theta }}_{c}^{\alpha }\right)J_{\alpha }\left({\hat{\theta }}_{c}^{\alpha }\right)H_{\alpha }^{-1}\left({\hat{\theta }}_{c}^{\alpha }\right)\right),$$

where $H_{\alpha }\left(\theta \right)$ and $J_{\alpha }\left(\theta \right)$ are given in (11) and (12). The optimum α will be the one that minimizes expression (16). The choice of the pilot estimator is probably one of the major drawbacks of this approach, as it may lead to a choice of α too close to that used for the pilot estimator. A pilot estimator with α≈0.4, was proposed in [13] after some simulations, in concordance with [30], where the initial choice of a pilot is suggested to be a robust one in order to obtain the best results in terms of robustness.

### 5.2. Iris Data

The Iris data (Fisher, [31]) includes 3 categories of 50 sample values each, where each category refers to a type of iris plant: *setosa*, *versicolor* and *virginica*. Each plant is categorized in its class and described by other 4 variables: (1) sepal length, (2) sepal width, (3) petal length and (4) petal width. This is one of the most known data sets for discriminant analysis. [32] proposed the use of a Gaussian finite mixture for modeling Iris data, in which each known class is modeled by a single Gaussian term with the same variance-covariance matrix. The resulting model is as follows

(17) $$\begin{array}{c} f\left(x\right)=\frac{1}{3}N\left(\mu_{1},\Sigma \right)+\frac{1}{3}N\left(\mu_{2},\Sigma \right)+\frac{1}{3}N\left(\mu_{3},\Sigma \right), \end{array}$$

with

$$\mu_{1}={\left(\mu_{11},\mu_{12},\mu_{13},\mu_{14}\right)}^{T},\;\mu_{2}={\left(\mu_{21},\mu_{22},\mu_{23},\mu_{24}\right)}^{T},\;\mu_{3}={\left(\mu_{31},\mu_{32},\mu_{33},\mu_{34}\right)}^{T}$$

and

$$\begin{array}{c} \Sigma =\left(\begin{array}{cccc} \sigma_{1}^{2} & \sigma_{12} & \sigma_{13} & \sigma_{14}\\ \sigma_{21} & \sigma_{2}^{2} & \sigma_{23} & \sigma_{24}\\ \sigma_{31} & \sigma_{32} & \sigma_{3}^{2} & \sigma_{34}\\ \sigma_{41} & \sigma_{42} & \sigma_{43} & \sigma_{4}^{2} \end{array}\right). \end{array}$$

Exact values can be obtained by *MclustDA*() function of *mclust* package in R Software ([32]).

We propose a composite likelihood approach to modeling (17) where we suppose independence between the two first and two last variables. This is

(18) $$f_{CL}\left(y\right)=\frac{1}{3}CLN_{1}+\frac{1}{3}CLN_{2}+\frac{1}{3}CLN_{3},$$

with

$$\begin{array}{c} CLN_{i}=f_{A_{i1}}^{N}\left(\rho_{12},y\right)f_{A_{i2}}^{N}\left(\rho_{34},y\right), \end{array}$$

where $f_{A_{i1}}^{N}\left(\rho_{12},y\right)=f_{A_{i1}}^{N}\left(\rho_{12},\mu_{i1},\mu_{i2},\Sigma_{A_{1}},y\right)$ and $f_{A_{i2}}^{N}\left(\rho_{34},y\right)=f_{A_{i2}}^{N}\left(\rho_{34},\mu_{i3},\mu_{i4},\Sigma_{A_{2}},y\right)$, i=1,2,3 are bivariate normals with variance-covariance matrices

$$\begin{array}{c} \Sigma_{A_{1}}=\left(\begin{array}{cc} \sigma_{1}^{2} & \rho_{12}\sigma_{1}\sigma_{2}\\ \rho_{12}\sigma_{1}\sigma_{2} & \sigma_{2}^{2} \end{array}\right),\;\Sigma_{A_{2}}=\left(\begin{array}{cc} \sigma_{3}^{2} & \rho_{34}\sigma_{3}\sigma_{4}\\ \rho_{34}\sigma_{3}\sigma_{4} & \sigma_{4}^{2} \end{array}\right). \end{array}$$

We are going to evaluate the behavior of the CLDIC criterion proposed in previous sections. After estimating parameters $\rho_{12}$ and $\rho_{34}$ in (18), we consider 10 different subsets of the IRIS data:

- SE subset: 50 first observations, corresponding to Setosa plants (n=50).
- VE subset: 50 second observations, corresponding to Versicolor plants (n=50).
- VI subset: 50 last observations, corresponding to Virginica plants (n=50).
- SE(VE) subset: SE subset with 2 first observations of VE subset (n=52).
  Equivalently: SE(VI), VE(SE), VE(VI), VI(SE) and VI(VE).
- VI(SE+VE) subset: VI subset with 2 first observations of SE and VE subsets (n=54).

In Table 4, chosen models for each one of the subsets are obtained by the proposed CLDIC criterion. When a “pure” subset is considered, all the tuning parameters lead to optimal decisions, but when a “contaminated” subset is under consideration, only α=0.2,0.3 have an optimal response in all the cases.

We now apply the ad hoc approach presented in Section 5.1 for selecting the tuning parameter α in a composite likelihood framework. Applying this procedure to our data set though a grid search of length 100 and by means of a pilot estimator with α=0.4 leads to the optimal tuning parameter α=0.22, what is in concordance with the obtained results (see Table 5). We can see that the use of other pilot estimators would not affect very much to the final decission.

### 5.3. Wine Data

We now work with Wine data ([33]), which contain a chemical analysis of 178 Italian wines from three different cultivars (Barolo, Grignolino, Barbera) yielded 13 measurements. In order to illustrate our criterion, we will work with only first four explanatory variables: Alcohol, Malic, Ash and Alkalinity. As in the previous section, we adjust a Gaussian mixture model with weights, in this case: 59/178, 72/178 and 47/178 corresponding to Barolo, Grignolino and Barbera classes, respectively. We now consider these 10 different subsets of the Wine data:

- BO subset: 20 first observations of Barolo wines (n=20).
- GR subset: 20 first observations of Grignolino wines (n=20).
- BA subset: 20 first observations of Barbera wines (n=20).
- BO(GR) subset: BO subset with 5 first observations of GR subset (n=25).
  Equivalently: BO(BA), GR(BO), GR(BA), BA(BO) and BA(GR).
- BA(BO+GR) subset: BA subset with 3 first observations of BO and GR subsets (n=26).

We can observe how, for medium values of α, the discrimination is perfect (see Table 6). Applying ad-hoc tuning parameter choice procedure we obtain $\alpha_{opt}\approx 0.51$, with a perfect discrimination again (Table 5).

## 6. Conclusions and Future Research

In this paper, we have addressed the problem of model selection in the framework of composite likelihood methodology, on the basis of the DPD as a measure of the closeness of the composite density and the true model that drives the data. In this context, an information criterion is introduced and studied which is defined by means of composite minimum distance type estimators of the unknown parameters, well-known for having nice robustness properties. Thanks to a simulation study, we have shown that the proposed here model selection criterion works well in practice and mainly that the use of CMDPDE makes the criterion more robust than the criteria based on the classic CMLE and the Kullback–Leibler divergence, given in [22]. The analysis of two real data examples of the literature illustrate on how the model selection criterion, presented here, can be applied in practical cases. This paper is a part of a series of papers by the authors where composite likelihood ideas and methods are harmonically weaved with divergence theoretic methods in order to develop statistical inference (estimation and testing of hypotheses) and model selection criteria, as well. We envision future work in some directions. The development of change point methodology on the basis of composite density with CMDPDE and divergence measures would be maybe an appealing problem for a future research on the topic. However, all the information theoretic methods developed on the basis of the composite likelihood depend on the choice of the family of sets ${\left\{A_{k}\right\}}_{k=1}^{K}$, appeared in Formula (1). A question is raised at this point: how the information theoretic procedures developed on the basis of the composite likelihood are affected by this family of sets? It is an appealing problem which deserves also investigation in a future work.

## Figures and Tables

**Table 1:** Main results, Scenario 1a.

	α=0 (CAIC)							α=0.3						
ω	0	0.25	0.45	0.5	0.55	0.75	1	0	0.25	0.45	0.5	0.55	0.75	1
ν=5,ρ=−0.15														
n = 5	0	1	269	499	713	996	1000	0	0	273	498	712	1000	1000
7	0	1	246	504	758	998	1000	0	1	220	511	738	999	1000
10	0	0	202	482	775	1000	1000	0	0	185	467	771	1000	1000
20	0	0	114	486	871	1000	1000	0	0	112	473	866	1000	1000
40	0	0	41	459	947	1000	1000	0	0	54	496	954	1000	1000
50	0	0	21	475	964	1000	1000	0	0	41	556	986	1000	1000
70	0	0	9	461	985	1000	1000	0	0	48	656	995	1000	1000
100	0	0	5	472	992	1000	1000	0	0	142	885	1000	1000	1000
ν=10,ρ=−0.15														
5	0	3	222	445	688	996	1000	0	3	218	433	688	997	1000
7	0	1	191	439	720	1000	1000	0	0	179	431	690	999	1000
10	0	0	163	432	747	1000	1000	0	0	152	402	725	1000	1000
20	0	0	59	399	819	1000	1000	0	0	49	361	773	1000	1000
40	0	0	19	336	912	1000	1000	0	0	12	326	899	1000	1000
50	0	0	6	362	936	1000	1000	0	0	10	334	925	1000	1000
70	0	0	1	292	960	999	1000	0	0	2	356	973	1000	1000
100	0	0	0	301	983	1000	1000	0	0	1	531	992	1000	1000
ν=30,ρ=−0.15														
5	0	4	237	423	677	997	1000	0	2	235	413	656	996	1000
7	0	0	155	394	689	1000	1000	0	0	141	379	677	999	1000
10	0	0	144	413	719	1000	1000	0	0	134	393	701	1000	1000
20	0	0	57	351	801	1000	1000	0	0	40	311	764	1000	1000
40	0	0	11	296	904	1000	1000	0	0	8	263	882	1000	1000
50	0	0	6	271	918	1000	1000	0	0	3	253	903	1000	1000
70	0	0	1	225	942	1000	1000	0	0	0	229	941	1000	1000
100	0	0	0	208	978	1000	1000	0	0	0	303	989	1000	1000
ν=10,ρ=−0.10														
5	0	4	242	464	680	996	1000	0	3	238	459	682	999	1000
7	0	0	187	461	733	997	1000	0	0	199	457	731	998	1000
10	0	0	162	445	738	1000	1000	0	0	165	407	713	1000	1000
20	0	0	62	378	807	1000	1000	0	0	59	354	789	1000	1000
40	0	0	19	357	902	999	1000	0	0	14	333	895	1000	1000
50	0	0	6	325	932	1000	1000	0	0	8	325	931	1000	1000
70	0	0	2	305	954	1000	1000	0	0	6	367	967	1000	1000
100	0	0	0	307	979	1000	1000	0	0	2	507	993	1000	1000
ν=10,ρ=0.10														
5	0	11	268	459	669	991	1000	1	11	268	478	680	993	1000
7	0	1	211	456	720	999	1000	0	3	207	464	716	998	1000
10	0	0	168	423	704	1000	1000	0	0	162	403	702	1000	1000
20	0	0	86	360	789	1000	999	0	0	89	357	786	1000	1000
40	0	0	35	367	893	1000	1000	0	0	38	398	896	1000	1000
50	0	0	19	331	886	1000	1000	0	0	19	360	913	1000	1000
70	0	0	11	311	933	1000	1000	0	0	16	379	963	1000	1000
100	0	0	2	276	969	1000	1000	0	0	7	490	985	1000	1000

**Table 2:** Main results, Scenario 1b.

	α=0 (CAIC)				α=0.2				α=0.4			
	0	0.25	0.75	1	0	0.25	0.75	1	0	0.25	0.75	1
n = 40	0	0	39	731	0	0	537	961	0	0	580	949
50	0	0	24	732	0	0	859	990	0	0	944	994
60	0	0	14	772	0	0	999	1000	0	1	999	1000
70	0	0	9	734	0	0	999	1000	0	27	999	1000
80	0	0	5	770	0	1	1000	1000	0	326	1000	1000
90	0	0	4	782	0	23	1000	1000	2	794	1000	1000
100	0	0	4	802	0	173	1000	1000	26	978	1000	1000

**Table 3:** Main results, Scenario 2.

	α=0 (CAIC)			α=0.3			α=0.5			α=0.7		
Model ${}^{*}$	N1	N2	MT	N1	N2	MT	N1	N2	MT	N1	N2	MT
True model: $N(\mu_{1}^{N},\Sigma )$												
n = 5	957	24	19	950	16	34	939	23	38	936	28	36
7	970	19	11	966	13	24	961	13	26	950	22	28
10	993	3	4	986	4	10	979	6	15	971	6	23
20	1000	0	0	1000	0	0	998	0	2	997	0	3
40	1000	0	0	1000	0	0	1000	0	0	1000	0	0
50	1000	0	0	1000	0	0	1000	0	0	1000	0	0
70	1000	0	0	1000	0	0	1000	0	0	1000	0	0
100	1000	0	0	1000	0	0	1000	0	0	999	0	0
True model: $N(\mu_{2}^{N},\Sigma )$												
5	29	638	333	34	610	356	38	639	323	50	646	304
7	15	622	363	13	589	398	17	599	384	28	627	345
10	6	610	384	5	540	455	5	540	455	11	586	403
20	1	612	387	1	518	481	1	472	527	1	527	472
40	0	566	434	0	650	350	0	590	410	0	614	386
50	0	561	439	0	804	196	0	797	203	0	835	165
70	0	584	416	0	987	13	0	994	6	0	998	2
100	0	520	480	0	1000	0	0	1000	0	0	1000	0
True model: $t_{\nu =10}\left(\mu^{t},\Sigma \right)$												
5	2	15	983	1	6	993	1	8	991	3	15	982
7	0	3	997	0	1	999	2	2	996	0	4	996
10	0	1	999	0	2	998	0	2	998	0	3	997
20	0	0	1000	0	0	1000	0	0	1000	0	0	1000
40	0	0	1000	0	0	1000	0	0	1000	0	0	1000
50	0	0	1000	0	0	1000	0	0	1000	0	0	1000
70	0	0	1000	0	0	1000	0	0	1000	0	0	1000
100	0	0	1000	0	0	1000	0	4	996	0	296	704
True model: $0.7N\left(\mu_{2}^{N},\Sigma \right)+0.3t_{\nu =10}\left(\mu^{t},\Sigma \right)$												
5	6	384	610	6	375	619	4	401	595	11	452	537
7	1	331	668	1	294	705	1	317	682	1	373	626
10	1	261	738	1	218	781	1	253	746	1	306	693
20	0	109	891	0	101	899	0	107	893	0	141	859
40	0	26	974	0	126	874	0	122	878	0	166	834
50	0	13	987	0	311	689	0	345	655	0	445	555
70	0	6	994	0	948	52	0	982	18	0	994	6
100	0	2	998	0	1000	0	0	1000	0	0	999	1
True model: $\frac{1}{3}N\left(\mu_{1}^{N},\Sigma \right)+\frac{1}{3}N\left(\mu_{2}^{N},\Sigma \right)+\frac{1}{3}t_{\nu =10}\left(\mu^{t},\Sigma \right)$												
5	127	377	496	121	363	516	107	392	501	107	424	469
7	87	357	556	70	339	591	66	356	578	63	396	541
10	69	326	605	61	314	625	56	330	614	45	381	574
20	37	259	704	25	298	677	17	337	646	15	349	636
40	7	145	848	9	452	539	4	508	488	1	469	530
50	2	122	876	5	744	251	3	814	183	3	853	144
70	0	99	901	4	996	0	4	996	0	4	996	0
100	0	36	964	355	645	0	645	355	0	856	144	0

${}^{*}$ Here the model candidates are expressed as N1, N2, MT to denote $N(\mu_{1}^{N},\Sigma )$, $N(\mu_{2}^{N},\Sigma )$ and $t_{10}\left(\mu^{t},\Sigma \right)$, respectively.

**Table 4:** Selected model in each of the subsets. Iris data.

α	SE	VE	VI	SE(VE)	SE(VI)	VE(SE)	VE(VI)	VI(SE)	VI(VE)	VI(SE+VE)
0 (CAIC)	CN1	CN2	CN3	CN1	CN1	CN1${}^{*}$	CN2	CN1${}^{*}$	CN3	CN3
0.2	CN1	CN2	CN3	CN1	CN1	CN2	CN2	CN3	CN3	CN3
0.3	CN1	CN2	CN3	CN1	CN1	CN2	CN2	CN3	CN3	CN3
0.4	CN1	CN2	CN3	CN1	CN1	CN2	CN2	CN1${}^{*}$	CN3	CN3
0.5	CN1	CN2	CN3	CN1	CN1	CN2	CN2	CN1${}^{*}$	CN3	CN3
0.8	CN1	CN2	CN3	CN1	CN1	CN2	CN2	CN1${}^{*}$	CN3	CN3
**0.22**	CN1	CN2	CN3	CN1	CN1	CN2	CN2	CN3	CN3	CN3

**Table 5:** Selected α for different pilot estimators, ad-hoc tuning parameter selection procedure. Iris and Wine data.

	$\alpha_{\mathrm{pilot}}$	0	0.1	0.2	0.3	0.4	0.5	0.6	0.7	0.8	0.9	1
Iris	$\alpha_{opt}$	0.31	0.17	0.20	0.21	**0.22**	0.23	0.24	0.24	0.25	0.25	0.25
Wine	$\alpha_{opt}$	0.45	0.46	0.47	0.49	**0.51**	0.53	0.55	0.56	0.56	0.56	0.57

**Table 6:** Selected model in each of the subsets. Wine data.

α	BO	GR	BA	BO(GR)	BO(BA)	GR(BO)	GR(BA)	BA(BO)	BA(GR)	BA(BO+GR)
0 (CAIC)	CN1	CN2	CN3	CN1	CN1	CN2	CN2	CN3	CN3	CN2${}^{*}$
0.2	CN1	CN2	CN3	CN1	CN1	CN2	CN2	CN3	CN3	CN3
0.3	CN1	CN2	CN3	CN1	CN1	CN2	CN2	CN3	CN3	CN3
0.4	CN1	CN2	CN3	CN1	CN1	CN2	CN2	CN3	CN3	CN3
0.5	CN1	CN2	CN3	CN1	CN1	CN2	CN2	CN3	CN3	CN3
0.8	CN1	CN2	CN3	CN1	CN1	CN2	CN2	CN2${}^{*}$	CN2${}^{*}$	CN3
**0.51**	CN1	CN2	CN3	CN1	CN1	CN2	CN2	CN3	CN3	CN3

## Abbreviations

The following abbreviations are used in this manuscript:

MLE	Maximum likelihood estimator
CMLE	Composite maximum likelihood estimator
CLDIC	Composite likelihood DIC
DPD	Density power divergence
MDPDE	Minimum density power divergence estimator
CMDPDE	Composite minimum density power divergence estimator
AIC	Akaike Information Criterion
CAIC	Composite Akaike Information Criterion
TIC	Takeuchi Information Criterion

## Appendix A. Proof of Theorem 1

**Proof.** A Taylor expansion of $W_{\alpha }\left(\theta \right)$ around the true parameter $\theta_{0}$ and evaluated in $\theta ={\hat{\theta }}_{c}^{\alpha }$, gives

$$\begin{array}{cc} W_{\alpha }\left({\hat{\theta }}_{c}^{\alpha }\right) & =W_{\alpha }\left(\theta_{0}\right)+{\left(\frac{\partial W_{\alpha }\left(\theta \right)}{\partial \theta }\right)}_{\theta =\theta_{0}}\left({\hat{\theta }}_{c}^{\alpha }-\theta_{0}\right)\\ \; & +\frac{1}{2}{\left({\hat{\theta }}_{c}^{\alpha }-\theta_{0}\right)}^{T}{\left(\frac{\partial^{2}W_{\alpha }\left(\theta \right)}{\partial \theta \partial \theta^{T}}\right)}_{\theta =\theta_{0}}\left({\hat{\theta }}_{c}^{\alpha }-\theta_{0}\right)+o\left({∥\left({\hat{\theta }}_{c}^{\alpha }-\theta_{0}\right)∥}^{2}\right). \end{array}$$

Now,

$$\begin{array}{cc} \frac{\partial W_{\alpha }\left(\theta \right)}{\partial \theta } & =\int_{R^{m}}\left(1+\alpha \right)\mathrm{CL}{\left(\theta ,y\right)}^{\alpha }\frac{\partial \mathrm{CL}(\theta ,y)}{\partial \theta }dy-\left(1+\frac{1}{\alpha }\right)\alpha \int_{R^{m}}\mathrm{CL}{\left(\theta ,y\right)}^{\alpha -1}\frac{\partial \mathrm{CL}(\theta ,y)}{\partial \theta }g\left(y\right)dy\\ \; & =\left(1+\alpha \right)\int_{R^{m}}\mathrm{CL}{\left(\theta ,y\right)}^{\alpha +1}u\left(\theta ,y\right)dy-\left(1+\alpha \right)\int_{R^{m}}\mathrm{CL}{\left(\theta ,y\right)}^{\alpha }u\left(\theta ,y\right)g\left(y\right)dy. \end{array}$$

It is clear that if the true distribution *g* belongs to the parameter family f(.;θ),θ∈Θ and $\theta_{0}$ denotes the true value of the parameter θ, we get

$${\left(\frac{\partial W_{\alpha }\left(\theta \right)}{\partial \theta }\right)}_{\theta =\theta_{0}}=0.$$

Now we are going to get

$$\begin{array}{cc} \frac{\partial^{2}W_{\alpha }\left(\theta \right)}{\partial \theta \partial \theta^{T}} & =\left(1+\alpha \right)\left\{\int_{R^{m}}\left(1+\alpha \right)\mathrm{CL}{\left(\theta ,y\right)}^{\alpha +1}u\left(\theta ,y\right)u{\left(\theta ,y\right)}^{T}dy\right.\\ \; & \left.-\int_{R^{m}}\mathrm{CL}{\left(\theta ,y\right)}^{\alpha +1}\left(-\frac{\partial^{2}\log\mathrm{CL}\left(\theta ,y\right)}{\partial \theta \partial \theta^{T}}\right)dy\right.\\ \; & \left.-\alpha \int_{R^{m}}\mathrm{CL}{\left(\theta ,y\right)}^{\alpha }u\left(\theta ,y\right)u{\left(\theta ,y\right)}^{T}g\left(y\right)dy+\int_{R^{m}}\mathrm{CL}{\left(\theta ,y\right)}^{\alpha }\left(-\frac{\partial^{2}\log\mathrm{CL}\left(\theta ,y\right)}{\partial \theta \partial \theta^{T}}\right)g\left(y\right)dy\right\}. \end{array}$$

If the true distribution *g* belongs to the parameter family $f_{\theta }\left(\cdot ;\theta \right)$, θ∈Θ and $\theta_{0}$ denotes the true value of the parameter θ, verifies,

$$\begin{array}{cc} {\left(\frac{\partial^{2}W_{\alpha }\left(\theta \right)}{\partial \theta \partial \theta^{T}}\right)}_{\theta =\theta_{0}} & =\left(1+\alpha \right)\int_{R^{m}}\mathrm{CL}{\left(\theta_{0},y\right)}^{\alpha +1}u\left(\theta_{0},y\right)u{\left(\theta_{0},y\right)}^{T}dy\\ & =\left(1+\alpha \right)H_{\alpha }\left(\theta_{0}\right). \end{array}$$

Therefore,

$$nW_{\alpha }\left({\hat{\theta }}_{c}^{\alpha }\right)=nW_{\alpha }\left(\theta_{0}\right)+\frac{\left(1+\alpha \right)}{2}\sqrt{n}{\left({\hat{\theta }}_{c}^{\alpha }-\theta_{0}\right)}^{T}H_{\alpha }\left(\theta_{0}\right)\sqrt{n}\left({\hat{\theta }}_{c}^{\alpha }-\theta_{0}\right)+no\left({∥\left({\hat{\theta }}_{c}^{\alpha }-\theta_{0}\right)∥}^{2}\right).$$

But

$$\sqrt{n}\left({\hat{\theta }}_{c}^{\alpha }-\theta_{0}\right)\underset{n\to \infty }{\overset{L}{\to }}N\left(0,H_{\alpha }{\left(\theta_{0}\right)}^{-1}J_{\alpha }\left(\theta_{0}\right)H_{\alpha }{\left(\theta_{0}\right)}^{-1}\right),$$

and $no\left({∥\left({\hat{\theta }}_{c}^{\alpha }-\theta_{0}\right)∥}^{2}\right)=o\left(O_{p}\left(1\right)\right)=o_{p}\left(1\right).$ The asymptotic distribution of the quadratic form $\sqrt{n}{\left({\hat{\theta }}_{c}^{\alpha }-\theta_{0}\right)}^{T}H_{\alpha }\left(\theta_{0}\right)\sqrt{n}\left({\hat{\theta }}_{c}^{\alpha }-\theta_{0}\right),$ verifies

$$\sqrt{n}{\left({\hat{\theta }}_{c}^{\alpha }-\theta_{0}\right)}^{T}H_{\alpha }\left(\theta_{0}\right)\sqrt{n}\left({\hat{\theta }}_{c}^{\alpha }-\theta_{0}\right)\underset{n\to \infty }{\overset{L}{⟶}}\sum_{r=1}^{k}\lambda_{r}Z_{r}^{2}$$

being $\lambda_{r},$r=1,…,k, the eigenvalues of the matrix

$$H_{\alpha }\left(\theta_{0}\right)H_{\alpha }{\left(\theta_{0}\right)}^{-1}J_{\alpha }\left(\theta_{0}\right)H_{\alpha }{\left(\theta_{0}\right)}^{-1}=J_{\alpha }\left(\theta_{0}\right)H_{\alpha }{\left(\theta_{0}\right)}^{-1}$$

and $Z_{r}$ are independent normal random variable of mean zero and variance 1. Therefore,

$$\begin{array}{cc} E_{Y_{1},\ldots ,Y_{n}}\left[\sqrt{n}{\left({\hat{\theta }}_{c}^{a}-\theta_{0}\right)}^{T}H_{\alpha }\left(\theta_{0}\right)\sqrt{n}\left({\hat{\theta }}_{c}^{a}-\theta_{0}\right)\right] & =\sum_{r=1}^{k}\lambda_{r}+o_{p}\left(1\right)\\ & =trace\left(J_{\alpha }\left(\theta_{0}\right)H_{\alpha }{\left(\theta_{0}\right)}^{-1}\right)+o_{p}\left(1\right) \end{array}$$

and

$$E_{Y_{1},\ldots ,Y_{n}}\left[nW_{\alpha }\left({\hat{\theta }}_{c}^{\alpha }\right)\right]=nW_{\alpha }\left(\theta_{0}\right)+\frac{\left(1+\alpha \right)}{2}trace\left(J_{\alpha }\left(\theta_{0}\right)H_{\alpha }{\left(\theta_{0}\right)}^{-1}\right)+o_{p}\left(1\right).$$

Now a Taylor expansion of $W_{n,\alpha }\left(\theta \right)$, around ${\hat{\theta }}_{c}^{\alpha }$ and evaluated at $\theta =\theta_{0}$ gives

$$\begin{array}{cc} W_{n,\alpha }\left(\theta_{0}\right) & =W_{n,\alpha }\left({\hat{\theta }}_{c}^{\alpha }\right)+{\left(\frac{H_{n,\alpha }\left(\theta \right)}{\partial \theta }\right)}_{\theta ={\hat{\theta }}_{c}^{\alpha }}\left(\theta_{0}-{\hat{\theta }}_{c}^{\alpha }\right)\\ \; & +\frac{1}{2}{\left(\theta_{0}-{\hat{\theta }}_{c}^{\alpha }\right)}^{T}{\left(\frac{\partial^{2}W_{n,\alpha }\left(\theta \right)}{\partial \theta }\partial \theta^{T}\right)}_{\theta ={\hat{\theta }}_{c}^{\alpha }}\left(\theta_{0}-{\hat{\theta }}_{c}^{\alpha }\right)+o\left({∥\theta_{0}-{\hat{\theta }}_{c}^{\alpha }∥}^{2}\right). \end{array}$$

But

$$\begin{array}{cc} \frac{W_{n,\alpha }\left(\theta \right)}{\partial \theta } & =\left(\alpha +1\right)\int_{R^{m}}\mathrm{CL}{\left(\theta ,y\right)}^{\alpha +1}u\left(\theta ,y\right)dy-\left(\alpha +1\right)\frac{1}{n}\sum_{k=1}^{n}\mathrm{CL}{\left(\theta ,y_{k}\right)}^{\alpha }u\left(\theta ,y_{k}\right) \end{array}$$

therefore

$${\left(\frac{W_{n,\alpha }\left(\theta \right)}{\partial \theta }\right)}_{\theta ={\hat{\theta }}_{c}^{a}}\underset{n\to \infty }{\overset{P}{\to }}0.$$

On the other hand

$$\begin{array}{cc} \frac{\partial^{2}W_{n,\alpha }\left(\theta \right)}{\partial \theta \;\partial \theta^{T}} & =\left(1+\alpha \right)\left\{\int_{R^{m}}\left(1+\alpha \right)\mathrm{CL}{\left(\theta ,y\right)}^{\alpha +1}u{\left(\theta ,y\right)}^{T}u\left(\theta ,y\right)dy+\int_{R^{m}}\mathrm{CL}{\left(\theta ,y\right)}^{\alpha +1}\frac{\partial u\left(\theta ,y\right)}{\partial \theta^{T}}dy\right.\\ \; & \left.-\frac{1}{n}\sum_{i=1}^{n}\alpha \mathrm{CL}{\left(\theta ,y_{i}\right)}^{\alpha }u{\left(\theta ,y_{i}\right)}^{T}u\left(\theta ,y_{i}\right)-\frac{1}{n}\sum_{i=1}^{n}\mathrm{CL}{\left(\theta ,y_{i}\right)}^{\alpha }\frac{\partial u\left(\theta ,y_{i}\right)}{\partial \theta^{T}}\right\}. \end{array}$$

But

$$\frac{1}{n}\sum_{i=1}^{n}\mathrm{CL}{\left(\theta ,y_{i}\right)}^{\alpha }u{\left(\theta ,y_{i}\right)}^{T}u\left(\theta ,y_{i}\right)\underset{n\to \infty }{\overset{P}{\to }}\int_{R^{m}}\mathrm{CL}{\left(\theta ,y\right)}^{\alpha +1}u{\left(\theta ,y\right)}^{T}u\left(\theta ,y\right)dy$$

and

$$\frac{1}{n}\sum_{i=1}^{n}\mathrm{CL}{\left(\theta ,y_{i}\right)}^{\alpha }\frac{\partial u\left(\theta ,y_{i}\right)}{\partial \theta^{T}}\underset{n\to \infty }{\overset{P}{\to }}\int_{R^{m}}\mathrm{CL}{\left(\theta ,y\right)}^{\alpha +1}\frac{\partial u\left(\theta ,y\right)}{\partial \theta^{T}}dy.$$

Therefore

$${\left(\frac{\partial^{2}H_{n,\alpha }\left(\theta \right)}{\partial \theta \partial \theta^{T}}\right)}_{\theta ={\hat{\theta }}_{c}^{\alpha }}\underset{n\to \infty }{\overset{P}{\to }}\left(1+\alpha \right)H_{\alpha }\left(\theta_{0}\right).$$

We can now write

$$nW_{n,\alpha }\left(\theta_{0}\right)=nW_{n,\alpha }\left({\hat{\theta }}_{c}^{\alpha }\right)+\frac{\left(1+\alpha \right)}{2}\sqrt{n}{\left(\theta_{0}-{\hat{\theta }}_{c}^{\alpha }\right)}^{T}H_{\alpha }\left(\theta_{0}\right)\sqrt{n}\left(\theta_{0}-{\hat{\theta }}_{c}^{\alpha }\right)+o_{p}\left(1\right).$$

It is clear that

$$\begin{array}{cc} E_{Y_{1},\ldots ,Y_{n}}\left[\sqrt{n}{\left(\theta_{0}-{\hat{\theta }}_{c}^{\alpha }\right)}^{T}H_{\alpha }\left(\theta_{0}\right)\sqrt{n}\left(\theta_{0}-{\hat{\theta }}_{c}^{\alpha }\right)\right] & =\sum_{r=1}^{k}\lambda_{r}+o_{p}\left(1\right)\\ \; & =trace\left(J_{\alpha }\left(\theta_{0}\right)H_{\alpha }{\left(\theta_{0}\right)}^{-1}\right)+o_{p}\left(1\right). \end{array}$$

Then

$$E_{Y_{1},\ldots ,Y_{n}}\left[nW_{n,\alpha }\left(\theta_{0}\right)\right]=E_{Y_{1},\ldots ,Y_{n}}\left[nW_{n,\alpha }\left({\hat{\theta }}_{c}^{\alpha }\right)\right]+\frac{\left(1+\alpha \right)}{2}trace\left(J_{\alpha }\left(\theta_{0}\right)H_{\alpha }{\left(\theta_{0}\right)}^{-1}\right)+o_{p}\left(1\right)$$

and, on the other hand, it is clear that

$$E_{Y_{1},\ldots ,Y_{n}}\left[W_{n,\alpha }\left(\theta_{0}\right)\right]=W_{\alpha }\left(\theta_{0}\right).$$

Therefore,

$$\begin{array}{cc} E_{Y_{1},\ldots ,Y_{n}}\left[nW_{\alpha }\left({\hat{\theta }}_{c}^{\alpha }\right)\right] & =nW_{\alpha }\left(\theta_{0}\right)+\frac{\left(1+\alpha \right)}{2}trace\left(J_{\alpha }\left(\theta_{0}\right)H_{\alpha }{\left(\theta_{0}\right)}^{-1}\right)+o_{p}\left(1\right)\\ \; & =E_{Y_{1},\ldots ,Y_{n}}\left[nW_{n,\alpha }\left(\theta_{0}\right)\right]+\frac{\left(1+\alpha \right)}{2}trace\left(J_{\alpha }\left(\theta_{0}\right)H_{\alpha }{\left(\theta_{0}\right)}^{-1}\right)+o_{p}\left(1\right)\\ \; & =E_{Y_{1},\ldots ,Y_{n}}\left[nW_{n,\alpha }\left({\hat{\theta }}_{c}^{\alpha }\right)\right]+\frac{\left(1+\alpha \right)}{2}trace\left(J_{\alpha }\left(\theta_{0}\right)H_{\alpha }{\left(\theta_{0}\right)}^{-1}\right)\\ \; & \;+\frac{\left(1+\alpha \right)}{2}trace\left(J_{\alpha }\left(\theta_{0}\right)H_{\alpha }{\left(\theta_{0}\right)}^{-1}\right)+o_{p}\left(1\right)\\ \; & =E_{Y_{1},\ldots ,Y_{n}}\left[nW_{n,\alpha }\left({\hat{\theta }}_{c}^{\alpha }\right)\right]+\left(1+\alpha \right)trace\left(J_{\alpha }\left(\theta_{0}\right)H_{\alpha }{\left(\theta_{0}\right)}^{-1}\right)+o_{p}\left(1\right). \end{array}$$

Hence $nW_{n,\alpha }\left({\hat{\theta }}_{c}^{\alpha }\right)+\left(1+\alpha \right)trace\left(J_{\alpha }\left(\theta_{0}\right)H_{\alpha }{\left(\theta_{0}\right)}^{-1}\right)$ is an asymptotic unbiased estimator of

$$E_{Y_{1},\ldots ,Y_{n}}\left[nW_{\alpha }\left({\hat{\theta }}_{c}^{\alpha }\right)\right].$$

 □

## Appendix B. Computation of the CLDIC in Section 4.1

We have to compute

$$CLDIC\left(M_{k}\right)=W_{n,\alpha }\left(\hat{\rho }\right)+\frac{\alpha +1}{n}\frac{J_{\alpha }\left(\hat{\rho }\right)}{H_{\alpha }\left(\hat{\rho }\right)},$$

where

(A1) $$\begin{array}{cc} W_{n,\alpha }\left(\hat{\rho }\right) & =\int_{R^{4}}\mathrm{CL}{\left(\hat{\rho },y\right)}^{\alpha +1}dy-\left(1-\alpha^{-1}\right)\frac{1}{n}\sum_{i=1}^{n}\mathrm{CL}{\left(\hat{\rho },y_{i}\right)}^{\alpha }, \end{array}$$

(A2) $$\begin{array}{cc} J_{\alpha }\left(\hat{\rho }\right) & =\int_{R^{4}}\mathrm{CL}{\left(\hat{\rho },y\right)}^{2\alpha +1}u{\left(\hat{\rho },y\right)}^{2}dy-{\left(\int_{R^{4}}\mathrm{CL}{\left(\hat{\rho },y\right)}^{\alpha +1}u\left(\hat{\rho },y\right)dy\right)}^{2}, \end{array}$$

(A3) $$\begin{array}{cc} H_{\alpha }\left(\hat{\rho }\right) & =-\int_{R^{4}}\mathrm{CL}{\left(\hat{\rho },y\right)}^{\alpha +1}u{\left(\hat{\rho },y\right)}^{2}dy, \end{array}$$

for our candidate models, namely, composite normal and composite 4-variate *t*-distribution. As commented in Section 4.1, we consider a composite likelihood function based on the product of two bivariate distributions with common variance-covariance matrix. It is therefore, necessary in this example, to obtain values (A1), (A2) and (A3) for both composite normal and composite *t*-distributions. However, as stated in [10], while the sensitivity and variability matrices can be sometimes be evaluated explicitly, it is more usual to use empirical estimates. Following this comment, in the current example, we compute Equations (A1), (A2) and (A3) empirically through the sample data using

$$\begin{array}{cc} {\hat{W}}_{n,\alpha }\left(\hat{\rho }\right) & =\sum_{i=1}^{n}\mathrm{CL}{\left(\hat{\rho },y_{i}\right)}^{\alpha +1}-\left(1-\alpha^{-1}\right)\frac{1}{n}\sum_{i=1}^{n}\mathrm{CL}{\left(\hat{\rho },y_{i}\right)}^{\alpha },\\ {\hat{J}}_{\alpha }\left(\hat{\rho }\right) & =\sum_{i=1}^{n}\mathrm{CL}{\left(\hat{\rho },y_{i}\right)}^{2\alpha +1}u{\left(\hat{\rho },y_{i}\right)}^{2}-{\left(\sum_{i=1}^{n}\mathrm{CL}{\left(\hat{\rho },y_{i}\right)}^{\alpha +1}u\left(\hat{\rho },y_{i}\right)\right)}^{2}\\ {\hat{H}}_{\alpha }\left(\hat{\rho }\right) & =-\sum_{i=1}^{n}\mathrm{CL}{\left(\hat{\rho },y_{i}\right)}^{\alpha +1}u{\left(\hat{\rho },y_{i}\right)}^{2}. \end{array}$$

Now, we obtain the score of the composite likelihood $u(\hat{\rho },y_{i})$ explicitly for both cases. By equation (A.5) in [12],

$$\begin{array}{cc} u^{N}\left(\hat{\rho },y_{i}\right) & =\frac{\hat{\rho }}{1-{\hat{\rho }}^{2}}\left[2+\frac{1}{\hat{\rho }}\left(t_{1i}t_{2i}+t_{3i}t_{4i}\right)\right.\\ \; & \left.\;-\frac{1}{1-{\hat{\rho }}^{2}}\left(t_{1i}^{2}-2\hat{\rho }t_{1i}t_{2i}+t_{2i}^{2}\right)-\frac{1}{1-{\hat{\rho }}^{2}}\left(t_{3i}^{2}-2\hat{\rho }t_{3i}t_{4i}+t_{4i}^{2}\right)\right], \end{array}$$

with $t_{ji}=y_{ji}-\mu_{j}$, j=1,…,4. On the other hand, we want to compute $u^{t_{\nu }}\left(\hat{\rho },y_{i}\right)$.

$$\begin{array}{cc} u^{t_{\nu }}\left(\hat{\rho },y_{i}\right) & =\frac{\partial {\mathrm{CL}}^{t_{\nu }}\left(\hat{\rho },y_{i}\right)}{\partial \hat{\rho }}=\frac{\partial \log{\mathrm{CL}}^{t_{\nu }}\left(\hat{\rho },y_{i}\right)}{\partial \hat{\rho }}=\frac{1}{{\mathrm{CL}}^{t_{\nu }}\left(\hat{\rho },y_{i}\right)}\frac{\partial {\mathrm{CL}}^{t_{\nu }}\left(\hat{\rho },y_{i}\right)}{\partial \hat{\rho }}\\ \; & =\frac{1}{f_{12}^{t_{\nu }}\left(y_{i};\hat{\rho }\right)f_{34}^{t_{\nu }}\left(y_{i};\hat{\rho }\right)}\left[\frac{\partial }{\partial \hat{\rho }}f_{12}^{t_{\nu }}\left(y_{i};\hat{\rho }\right)f_{34}^{t_{\nu }}\left(y_{i};\hat{\rho }\right)\right]\\ \; & =\frac{1}{f_{12}^{t_{\nu }}\left(y_{i};\hat{\rho }\right)f_{34}^{t_{\nu }}\left(y_{i};\hat{\rho }\right)}\left[\left(\frac{\partial }{\partial \hat{\rho }}f_{12}^{t_{\nu }}\left(y_{i};\hat{\rho }\right)\right)f_{34}^{t_{\nu }}\left(y_{i};\hat{\rho }\right)+f_{12}^{t_{\nu }}\left(y_{i};\hat{\rho }\right)\left(\frac{\partial }{\partial \hat{\rho }}f_{34}^{t_{\nu }}\left(y_{i};\hat{\rho }\right)\right)\right]\\ \; & =\frac{1}{f_{12}^{t_{\nu }}\left(y_{i};\hat{\rho }\right)}\left(\frac{\partial }{\partial \hat{\rho }}f_{12}^{t_{\nu }}\left(y_{i};\hat{\rho }\right)\right)+\frac{1}{f_{34}^{t_{\nu }}\left(y_{i};\hat{\rho }\right)}\left(\frac{\partial }{\partial \hat{\rho }}f_{34}^{t_{\nu }}\left(y_{i};\hat{\rho }\right)\right). \end{array}$$

Now, it can be shown that

$$\begin{array}{c} \frac{\partial f_{12}^{t_{\nu }}\left(y_{i};\hat{\rho }\right)}{\partial \hat{\rho }}=f_{12}^{t_{\nu }}\left(y_{i};\hat{\rho }\right)\frac{\nu \left[\left(\nu -2\right){\hat{\rho }}^{3}-t_{1i}t_{2i}\nu {\hat{\rho }}^{2}+\left(\left(t_{1i}^{2}+t_{2i}^{2}-1\right)\nu +t_{2i}^{2}+t_{1i}^{2}+2\right)\hat{\rho }-t_{1i}t_{2i}\nu -2t_{1i}t_{2i}\right]}{\left(1-{\hat{\rho }}^{2}\right)\left[\left(\nu -2\right){\hat{\rho }}^{2}+2t_{1i}t_{2i}\hat{\rho }-\nu -t_{1i}^{2}-t_{2i}^{2}+2\right]} \end{array}$$

and

$$\begin{array}{c} \frac{\partial f_{34}^{t_{\nu }}\left(y_{i};\hat{\rho }\right)}{\partial \hat{\rho }}=f_{34}^{t_{\nu }}\left(y_{i};\hat{\rho }\right)\frac{\nu \left[\left(\nu -2\right){\hat{\rho }}^{3}-t_{3i}t_{4i}\nu {\hat{\rho }}^{2}+\left(\left(t_{3i}^{2}+t_{4i}^{2}-1\right)\nu +t_{4i}^{2}+t_{3i}^{2}+2\right)\hat{\rho }-t_{1i}t_{4i}\nu -2t_{3i}t_{4i}\right]}{\left(1-{\hat{\rho }}^{2}\right)\left[\left(\nu -2\right){\hat{\rho }}^{2}+2t_{3i}t_{4i}\hat{\rho }-\nu -t_{3i}^{2}-t_{4i}^{2}+2\right]}. \end{array}$$
